# Influence of Milk Pasteurization on the Key Aroma Compounds in a 30 Weeks Ripened Pilot-Scale Gouda Cheese Elucidated by the Sensomics Approach

**DOI:** 10.1021/acs.jafc.4c01813

**Published:** 2024-05-03

**Authors:** Philipp W. Duensing, Jörg Hinrichs, Peter Schieberle

**Affiliations:** † Former Chair for Food Chemistry, Faculty of Chemistry, Technical University of Munich, Lise-Meitner-Str. 34, D-85354 Freising, Germany; ‡ Department Soft Matter Science and Dairy Technology, Institute of Food Science and Biotechnology, University of Hohenheim, Garbenstraße 21, D-70599 Stuttgart, Germany

**Keywords:** gas chromatography/olfactometry, aroma extract dilution analysis, Gouda cheese, stable isotope dilution assay, aroma recombination, milk pasteurization

## Abstract

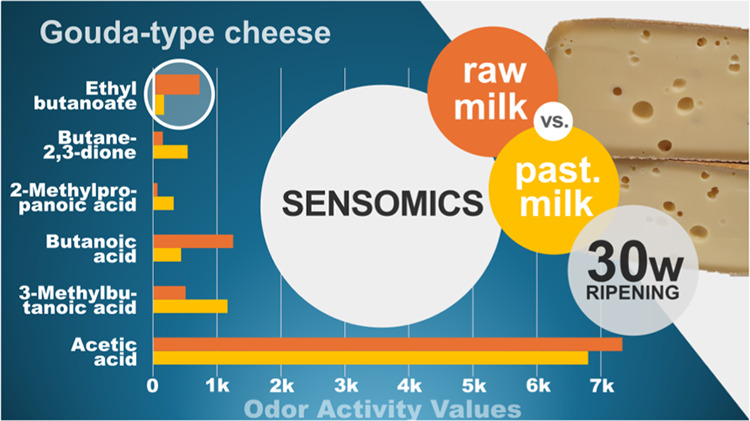

Gouda cheese was produced from pasteurized milk and ripened for 30 weeks (PM-G). By application of gas chromatography/olfactometry and an aroma extract dilution analysis on the volatiles isolated by extraction/SAFE distillation, 25 odor-active compounds in the flavor dilution (FD) factor range from 16 to 4096 were identified. Butanoic acid, 2- and 3-methylbutanoic acid, and acetic acid showed the highest FD factors, and 2-phenylethanol, δ-decalactone, and δ-dodecalactone were most odor-active in the neutral-basic fraction. Quantitations by stable isotope dilution assays followed by a calculation of odor activity values (OAVs) revealed acetic acid, 3-methylbutanoic acid, butanoic acid, and butane-2,3-dione with the highest OAVs. Finally, an aroma recombinate prepared based on the quantitative data well agreed with the aroma profile of the PM-G. In Gouda cheese produced from raw (nonpasteurized) milk (RM-G), qualitatively the same set of odor-active compounds was identified. However, higher OAVs of butanoic acid, hexanoic acid, and their corresponding ethyl esters were found. On the other hand, in the PM-G, higher OAVs for 3-methylbutanoic acid, 3-methylbutanol, 3-methylbutanal, and butane-2,3-dione were determined. The different rankings of these key aroma compounds clearly reflect the aroma differences of the two Gouda-type cheeses. A higher activity of lipase in the RM-G and higher amounts of free l-leucine in PM-G on the other side were responsible for the differences in the concentrations of some key aroma compounds.

## Introduction

Cheeses are among the most traditional fermented foods in the world, with a history of about 8000 years. Gouda cheese originates from The Netherlands and is today the main representative of Dutch-type cheeses. It is traditionally produced from bovine milk and brine before ripening for 1 to 20 months. Gouda is used as a general term for different cheeses produced following the Dutch manner, and the cheese exhibits a characteristic aroma when ripened for a longer time, i.e., 6 to 8 months. Scientists have been interested in elucidating Gouda aroma for decades. First studies, in particular on volatile acids, were done by Svensen and Iyer in the 1960s.^1,2^ Since then, more than 150 different volatile compounds have been reported in Gouda cheese. Many among them are acids, alcohols, aldehydes, ketones, lactones, and esters.^3−10^ The most comprehensive analytical work with special focus on lipid-derived volatiles in Gouda was done by Alewijn et al.^7−9^ Quantitation of Gouda volatiles was mainly done on a semiquantitative level using gas chromatography with flame ionization detector^2,5^ or gas chromatography with mass spectrometric detection, but often using only one internal standard.^2,6−10^ However, it is well accepted today that only volatiles interacting with the human odorant receptors do contribute to the aroma of a given food.^11,12^ Gas chromatography/olfactometry (GC/O) is a well-established method to select from a bulk of odorless volatiles those odor-active compounds potentially contributing to the overall food aroma.^13^ Inagaki et al.^14^ were among the first to apply GC/O on a distillate isolated from Gouda cheese. They identified or tentatively identified about 38 odor-active compounds. The authors highlighted the previously unknown 12-methyltridecanal and several homologous aldehydes as being important for the Gouda aroma. Jo et al. isolated the volatile fractions in a high amount of commercial Gouda cheeses by SPME and reported on the identification or tentative identification of more than 90 aroma-active compounds by application of GC/O.^15^ Odor-active volatiles reported were butane-2,3-dione, 2- and 3-methylbutanal, methylpropanal, methional, ethyl butanoate, acetic acid, butanoic acid, homofuraneol, δ-decalactone, and 2-isobutyl-3-methoxypyrazine.

Numerous studies have shown that the application of GC/O alone is not able to characterize the odorants, generating the overall aroma profile of foods. Sensomics, formerly called molecular sensory science,^11^ has successfully been applied to the key aroma compounds of other cheeses, such as Emmentaler,^16,17^ Camembert,^18,19^ and Swiss Gruyere cheese.^20,21^ However, the sensomics approach has not been applied in Gouda cheese research so far.^12^

The first aim of this study was, therefore, to characterize the key aroma compounds in a 30 weeks ripened pilot-scale Gouda cheese produced with pasteurized milk by application of the sensomics approach. The analysis follows a stepwise procedure, i.e., the characterization of odor-active compounds by aroma extract dilution analysis (AEDA), the quantitation of selected compounds by stable isotope dilution assays (SIDA), the calculation of odor activity values (ratio of concentration to odor thresholds) to identify the key aroma compounds, and finally the evaluation of an aroma recombinate.

A second aim was to investigate the influence of milk pasteurization on the differences in the set of key aroma compounds. The volatile fraction of cheeses is generated as a result of a complex reaction cascade decomposing lactose, lipids, and proteins by endogenous milk enzymes, enzymes of the starter cultures used, and in raw milk cheese, additional enzymes of the native milk microflora.^22^ However, to date, only quite general studies have been performed on the effect of milk pasteurization on the entire set of cheese volatiles. For example, the increased formation of short-chain free fatty acids has been shown for Swiss-type cheese or Cheddar cheeses.^23−26^ For Gouda-type cheese, the volatile fractions made from either pasteurized milk or raw milk differed in their composition.^8,10^ However, no systematic study on Gouda cheese involving human odorant perception is yet available.

Thus, the sensomics approach was subsequently applied to a 30 weeks ripened pilot-scale Gouda cheese produced under the same conditions, but made from raw (nonpasteurized) milk. A comparison of differences in the odor activity values of key aroma compounds should elucidate the molecular reasons for differences in the overall aroma profiles of both Goude cheese types. In addition, the roles of lipase activity and the concentrations of free amino acid as aroma compound precursors should be clarified.

## Materials and Methods

### Pilot-Scale Gouda Cheese Made from Raw (Nonpasteurized) or Pasteurized Milk

The cheese was produced in cooperation with the Dairy for Research and Training, University of Hohenheim, Germany. For this purpose, a batch of raw, nonpasteurized milk was standardized (fat content 3.5%) and divided into two equal volumes (180 L each). One half was pasteurized (72–74 °C, 30 s) before use, yielding the pasteurized milk Gouda (PM-G), and the other half was directly used for cheese production resulting in raw milk Gouda (RM-G). After addition of calcium chloride, lysozyme, and defined starter cultures, a preripening (35 °C, 35 min) of the milk was done. Acidification and the added lab enzyme induced protein coagulation (45 min) and the formation of a gel. The material was soon cut by so-called ‘harps’ to obtain a suspension of curd particles in the whey. One part of the whey was then replaced with water to wash the curd. This enhanced syneresis and further reduced the moisture content of the curd. The following drainage removed almost the whole liquid to obtain a wet granular bulk, which was compacted and then cut into blocks. The blocks were filled into cheese molds and gradually pressed in three steps up to 0.38 MPa. The unripened cheeses were salted by immersion in brine for 28 h. Finally, cheeses were coated with a vinyl acetate polymer. Ripening took place under controlled conditions in a climate chamber at 15 °C and 80–85% humidity (batch 1). As a control, the entire cheese production and ripening was repeated under the same conditions after one year (batch 2).

### Aroma Recombination

For recombination experiments, a commercial Mozzarella cheese (Galbani, EDEKA, Germany) was freeze-dried and used as an almost odorless cheese matrix.

### Chemicals

Reference compounds of the odorants identified were obtained from the commercial sources given in parentheses: acetic acid (Merck, Darmstadt, Germany); butane-2,3-dione, butanoic acid, γ-decalactone, δ-decalactone, γ-dodecalactone, δ-dodecalactone, ethyl butanoate, ethyl hexanoate, 5-ethyl-3-hydroxy-4-methyl-2(5*H*)-furanone, hexanoic acid, 3-hydroxy-4,5-dimethyl-2(5*H*)-furanone, 4-hydroxy-3-methoxybenzaldehyde (vanillin), 2-isopropyl-3-methoxypyrazine, 3-methylbutanal, 2-methylbutanoic acid, 3-methylbutanoic acid, 3-methylbutanol, 2-methylpropanoic acid, γ-nonalactone, (*E*)-2-nonenal, pentanoic acid, 2-phenylacetic acid and 2-phenylethanol (Sigma-Aldrich Chemie, Taufkirchen, Germany); and 4-ethyloctanoic acid (ABCR GmbH & Co. KG, Karlsruhe, Germany).

The following reference compounds were synthesized as previously described: (*Z*)-2-nonenal^27^ and *trans*-4,5-epoxy-(*E*)-2-decenal.^28^

### Isotopically Labeled Internal Standards

The following isotopically labeled standards were synthesized as previously reported: (^13^C_4_)-butane-2,3-dione,^29^ (^2^H_2_)-butanoic acid, (^2^H_2_)-δ-decalactone and (^2^H_2_)-δ-dodecalactone,^30^ (^2^H_3_)-ethyl butanoate,^31^ (^2^H_3_)-ethyl hexanoate and (^2^H_3_)-hexanoic acid,^32^ (^2^H_2_)-3-methylbutanal,^33^ (^2^H_2_)-3-methylbutanol and (^2^H_2_)-3-methylbutanoic acid,^34^ (^2^H_3_)-pentanoic acid,^35^ (^13^C_2_)-2-phenylethanol,^36^ (^2^H_3_)-acetic acid, and (^13^C_2_)-2-phenylacetic acid were purchased from Sigma-Aldrich Chemie, and (^2^H_7_)-2-methylpropanoic acid was from Merck.

Diethyl ether, sodium carbonate, sodium chloride, and anhydrous sodium sulfate were purchased from Merck. Liquid nitrogen was obtained from Linde (Munich, Germany). Diethyl ether was freshly distilled prior to use. l-Norleucine and 4-methylumbelliferyl butanoate were obtained from Sigma-Aldrich.

### Isolation of the Volatile Fraction; Separation into Acidic and Neutral-Basic Compounds

The cheese was cubed, frozen with liquid nitrogen, and then powdered using a commercial kitchen blender. After the addition of anhydrous sodium sulfate, the cheese powder (300 g) was extracted with diethyl ether (total volume 1.2 L) by vigorous stirring at room temperature for 120 min. The mixture was filtered through defatted cotton wool, and the residue was extracted twice with diethyl ether (200 mL). The organic phases, exhibiting an intense cheese-like aroma, were combined and the volatiles were isolated using the solvent-assisted flavor evaporation approach (SAFE).^37^ To separate the bulk of acidic volatiles (AF) from the neutral-basic volatiles (NBF), the distillate was extracted three times with aqueous sodium carbonate (0.5 mol/L, total volume of 450 mL). The combined aqueous solutions were adjusted to pH 2 with hydrochloric acid (1 mol/L) and reextracted with diethyl ether (3 × 100 mL) to obtain the acidic compounds (AF). The organic phases containing either the acidic or neutral/basic volatiles were concentrated to ∼200 μL each by means of a Vigreux column (60 cm × 1 cm i.d.) followed by microdistillation.^38^

### High-Resolution Gas Chromatography/Olfactometry (HRGC/O)

HRGC was performed by means of a Carlo Erba type Mega series 5160 gas chromatograph (Hofheim, Germany) with helium as the carrier gas at a flow rate of 2.2 mL/min. The samples were applied by cold-injection onto the following capillary columns: J&W Scientific DB-FFAP (30 m × 0.32 mm i.d; 0.25 μm film thickness) (Folsom); J&W Scientific DB-1701 (30 m × 0.25 mm i.d; 0.25 μm film thickness); Varian DB-5 (30 m × 0.25 mm i.d; 0.25 μm film thickness) (Darmstadt, Germany). After injection of the sample (1 μL) at 40 °C (held for 2 min isothermally), the oven temperature was raised to 190 °C by 6 °C/min, then raised to 230 °C by 12 °C/min and held for 10 min (DB-FFAP, DB-1701). Separation on the DB-5 stationary phase was performed as follows: Starting at 40 °C (held for 2 min isothermally), the oven temperature was raised by 8 °C/min to 240 °C and then held for 10 min isothermally.

For HRGC/O, the effluent of the capillary column was split 1:1 by volume using a Y-shaped glass splitter (Chrompack, Frankfurt, Germany) and two deactivated fused silica capillaries (each 60 cm × 0.2 mm i.d.), transferring the gas flow to a sniffing port and a flame ionization detector (FID). The sniffing port, a cylindrically shaped alumina device housing the capillary, was mounted on a detector base of the GC and was heated to 200 °C. During a GC/O run, trained panelists placed their nose closely above the top of the sniffing port (outlet on top covered with Teflon) and evaluated the odor of the chromatographic effluent. All detected odor qualities were marked with their retention time in the chromatogram using a flavor language developed by our group.^39^ The GC/O evaluations of the original extract and the dilutions 1:16 and 1:128 were performed by three panelists. Retention indices of the compounds were calculated from the retention times of *n*-alkanes by linear interpolation.

### Aroma Extract Dilution Analysis (AEDA)

Flavor dilution (FD) factors of the odor-active compounds in the fractions NBF and AF were determined by AEDA using the FFAP capillary column.^38^ Fractions were diluted stepwise using diethyl ether to obtain dilutions of 1:1, 1:2, 1:4, 1:8, and 1:16···1:4096 of the original extract. Each dilution was analyzed by HRGC/O (injection volume of 1 μL) until no odor-active compound could be detected. The FD factor represents the last dilution in which the odorant was still detectable.

### High-Resolution Gas Chromatography/Mass Spectrometry (HRGC/MS)

For compound identification, mass spectra of the analyte and the reference compound were recorded by means of a gas chromatograph 5890 series II (Hewlett-Packard, Waldbronn, Germany) connected to a sector field mass spectrometer MAT 95 (Finnigan, Bremen, Germany) using the capillary columns DB-FFAP and DB-5. For compound identification, mass spectra were generated in the MS/EI mode, recorded at 70 eV ionization energy, and in the chemical ionization mode (MS/CI) at 115 eV with isobutane as the reactant gas.

### Quantitation by Stable Isotope Dilution Assays (SIDA)

Depending on the concentrations of an odor-active compound determined in the preliminary experiments, powdered cheese samples (between 1 and 50 g) were used. The samples were spiked with defined amounts of each labeled internal standard. Then, anhydrous sodium sulfate and diethyl ether were added. The samples were stirred overnight for equilibration and finally filtered through defatted cotton wool. The volatile fraction and the internal standards were isolated by SAFE distillation, and the distillate was separated into the acidic and neutral-basic fractions by treatment with a sodium bicarbonate solution as described above.

For quantification, two different HRGC-MS systems were used. Nearly all acids were present in quite high concentrations, and these were quantitated by means of a gas chromatograph Varian GC 3800 (Darmstadt, Germany) coupled to a Varian ion trap mass spectrometer Saturn 2000 using the DB-FFAP capillary column. Quantitations of the neutral/basic compounds were performed by means of a two-dimensional Thermo Quest TDGC-MS system consisting of a gas chromatograph Trace 2000 series (Egelsbach, Germany) coupled to a gas chromatograph Varian GC 3800 using the DB-FFAP-capillary column in the first dimension and either the DB-FFAP or DB-5 capillary column in the second dimension. The respective elution volume containing both the selected odorant and the internal standard was transferred onto a cold trap (−100 °C) using the moving column stream switching system (MCCS) (Thermo, Dreieich, Germany) located in the first oven. By heating the trap was heated to 200 °C, the analyte and standard were transferred onto the second column, which was coupled to a Varian Saturn 2000 mass spectrometer. For quantitation, all mass spectra were recorded in the chemical ionization mode (MS/CI) with methanol as the reactant gas and using the temperature programs detailed above. For compound identification, mass spectra were also generated in MS/EI mode. For each compound, a calibration factor was determined by analyzing mixtures of defined amounts of the labeled and unlabeled compound in five different mass ratios (5:1, 3:1, 1:1, 1:3, and 1:5) by GC/MS. The response factors, calculated from the peak areas and the amounts of the labeled and unlabeled compound, are summarized in Table 1.

**Table 1:** Selected Ions (m/z) and Response Factors (RF) Used in the Stable Isotope Dilution Assays of 15 Odor-Active Compounds

compound	ion	labeled standard	ion	RF^a^
butane-2,3-dione	87	(^13^C_4_)-butane-2,3-dione	91	1.00
butanoic acid	89	(^2^H_2_)-butanoic acid	91	0.87
δ-decalactone	171	(^2^H_2_)-δ-decalactone	173	0.99
δ-dodecalactone	199	(^2^*H*_2_)-δ-dodecalactone	201	1.01
acetic acid	61	(^2^H_3_)-acetic acid	64	0.98
ethyl butanoate	117	(^2^H_3_)-ethyl butanoate	120	0.95
ethyl hexanoate	145	(^2^H_3_)-ethyl hexanoate	148	0.97
hexanoic acid	117	(^2^H_3_)-hexanoic acid	120	0.81
2-methylpropanoic acid	89	(^2^H_7_)-2-methylpropanoic acid	96	0.87
3-methylbutanal	69	(^2^H_2_)-3-methylbutanal	71	0.98
3-methylbutanol	71	(^2^H_2_)-3-methylbutanol	73	1.07
3-methylbutanoic acid	103	(^2^H_2_)-3-methylbutanoic acid	105	0.87
pentanoic acid	103	(^2^H_3_)-pentanoic acid	106	0.90
2-phenylacetic acid	137	(^13^C_2_)-2-phenylacetic acid	139	0.86
2-phenylethanol	105	(^13^C_2_)-2-phenylethanol	107	1.02

a MS response factor (MS/CI) determined by analyzing defined mixtures of the analyte and the internal standard.

Quantitation of butane-2,3-dione was performed in the volatile fraction isolated by solid phase micro extraction (SPME). Cheese powder (1 g) was spiked with carbon-13 labeled butane-2,3-dione and equilibrated for 1 h with saturated sodium chloride solution in a tightly sealed SPME vessel. After the volatile compound and the internal standard were adsorbed on an SPME fiber coated with a Carboxen phase (Supelco, Bellefonte), direct application into the TDGC-MS-System followed by thermal desorption at 250 °C was performed.

### Ratio of 2-Methylbutanoic Acid to 3-Methylbutanoic Acid

Both acids were not separated on the columns used, and therefore, the sum of both isomers was obtained by SIDA. The ratio of the amounts of 2- to 3-methylbutanoic acid was then calculated by means of the intensities (MS/EI) of the characteristic fragments *m*/*z* 74 (2-methylbutanoic acid) and *m*/*z* 60 (3-methylbutanoic acid). Six mixtures of both compounds (80:20, 60:40; 40:60; 20:80; 10:90:5:95) were analyzed under the same conditions to obtain a calibration line plotting the intensity ratio of *m*/*z* 60 over the sum of *m*/*z* 60 + *m*/*z* 74 against the amount of 3-methylbutanoic acid in the mixture.

### Determination of Odor Thresholds

Sensory analyses were performed in a sensory room equipped with single booths at 21 ± 1 °C. A minimum of 15 weekly trained panelists were recruited from the institute. For the determination of odor thresholds, deodorized sunflower oil was used as a matrix. The purity of each odorant was tested by HRGC/O before use. A defined amount of the respective compound was dissolved in 0.1 mL of ethanol, and reference solutions were prepared by adding the solution to 500 mL of the oil. Samples were shaken and diluted stepwise 1:3 (v/v) with the oil. An aliquot of 10 mL of each dilution was then filled into cylindrical Teflon bottles (height = 7 cm; inner diameter = 3.5 cm) with caps. Each test sample was evaluated in a series of triangle tests against two blank samples (odorless oil) in the order of decreasing concentrations. The odor thresholds were determined by judging whether the odorant’s attribute (recognition threshold) or only a difference (detection threshold) compared to the odorless matrix could be smelled.^39,40^

### Aroma Profile Analysis

In previous sessions, the following eight odor qualities, represented by the compounds given in parentheses, were chosen for the sensory evaluation of Gouda cheese and the aroma recombinates: coconut-like (δ-decalactone), sweaty-cheesy (3-methylbutanoic acid), sweaty-rancid (butanoic acid), vinegar-like (acetic acid), honey-like (2-phenylacetic acid), buttery (butane-2,3-dione), malty (3-methylbutanal), and fruity (ethyl butanoate). Intensities of the aroma descriptors were ranked on a seven-point scale in steps of 0.5 units from 0 (not perceivable) to 3 (strongly perceivable). The values judged by the panelists were averaged.

### Aroma Recombination Experiments

Gouda cheese powder was freshly prepared as described above. In parallel, a Gouda cheese model was prepared by adding an aqueous stock solution to a lyophilized, odorless Mozzarella cheese powder prepared from Galbani (EDEKA, Germany).^17^ The following aroma compounds were added in their actual concentrations determined in the Gouda cheese: acetic acid, butane-2,3-dione, butanoic acid, δ-decalactone, δ-dodecalactone, ethyl butanoate, ethyl hexanoate, hexanoic acid, 3-methylbutanal, 3-methylbutanol, 2-methylbutanoic acid, 3-methylbutanoic acid, 2-methylpropanoic acid, pentanoic acid, 2-phenylacetic acid, and 2-phenylethanol. The pH value of the stock solution was adjusted to 5.3, and the final water content (32%) of the model matched the Gouda cheese composition. In one session, the cheese aroma recombined, and in another session, the freshly prepared Gouda samples were presented to the sensory panel and judged by an aroma profile analysis. In a third session, the overall similarity of the Gouda aroma and the aroma recombinate was evaluated using a seven-point scale from 0 (no similarity) to 3 (very good similarity).

#### Determination of Free Amino Acids

Gouda cheese powder (5 g) was spiked with the internal standard l-norleucine dissolved in water (5 mL) and was homogenized with an Ultra-Turrax (IKA, Staufen, Germany) at 10^4^ rpm for 30 s. After lyophilization, the residue was defatted with *n*-pentane and the solvent was removed by air-drying in a fume hood. The residue was again suspended in water and centrifuged, and the supernatant was lyophilized again. The lyophilizate (0.1 g) was dissolved in acetate buffer (pH 2.2), filtered (0.45 μm), and analyzed by means of an amino acid analyzer (Eppendorf Biotronik LC 3000, Maintal, Germany) using postcolumn derivatization with ninhydrine.

#### Determination of Lipase Activity

Powdered Gouda cheese (1 g) was suspended in phosphate buffer (100 mL, 0.2 mol/L, pH 7.2) and homogenized for 60 s using an Ultra-Turrax (IKA, Staufen, Germany) at 10^4^ rpm. To separate the fat phase, the extract was centrifuged at 5000 rpm for 20 min at 15 °C. The fat-free extract (1.5 mL) was centrifuged at 15 °C and 12,000 rpm for 20 min. The supernatant (0.2 mL) was mixed with 0.2 mL of a solution of 4-methylumbelliferyl butanoate (26.2 nmol/mL) at 37 °C. After 60 min, the fluorescence (excitation 351, emission 447) was measured against the solvent using a HITACHI F-2000 spectrofluorometer (Krefeld, Germany). The lipolytic activity was calculated using a calibration curve with 4-methylumbelliferone.

#### Enzymatic Determination of l-Lactate and d-Lactate

Powdered Gouda cheese (0.5 to 1 g) was extracted for 30 min with hot water (60 °C), the fat was frozen out, and the suspension was filtered. An aliquot of the filtrate (0.1–1.0 mL) was directly used for the determination of l- and d-lactate using a commercial enzyme kit (r-biopharm AG, Darmstadt, Germany). In the presence of NAD^+^, l-lactate (or d-lactate) was oxidized to pyruvate by means of l-lactate dehydrogenase (or d-lactate dehydrogenase, respectively). The absorption of the increasing NADH/H^+^ concentration was measured in thermostated cells at 340 nm using a Shimadzu double-beam ultraviolet–visible (UV/vis) spectrophotometer UV-2401PC (Munich, Germany).

## Results and Discussion

### Identification of the Odor-Active Compounds in Gouda Cheese from Pasteurized Milk (PM-G)

The volatiles from Gouda cheese made with pasteurized milk (PM-G) were extracted with diethyl ether, followed by SAFE distillation. The distillate revealed the characteristic Gouda aroma when sniffed from a strip of filter paper and was subsequently separated into the neutral-basic (NBF) and the acidic volatiles (AF). By application of HRGC/O on both fractions, 13 aroma-active compounds were detected in the neutral-basic fraction, and 12 additional odor-active compounds were detected in the acidic fraction. Sniffing of serial dilutions revealed the highest FD factor of 4096 in the acidic fraction for two compounds, a sweaty-rancid (**8**) and a sweaty-cheesy smelling compound (**9**), followed by a vinegar-like compound (**4**) with an FD factor of 2048 (Table 2). In the neutral/basic fraction, flowery (**12**), coconut-like (**18**), leather-like (**19**), and peach-like (**22**) smelling compounds were detected with somewhat lower FD factors of 512 (Table 2).

**Table 2:** Most Odor-Active Compounds (FD ≥ 16) in 30 Weeks Ripened Gouda Cheese Made from Pasteurized Milk (PM-G)

				RI^d^				
no.	compound^a^	odor quality^b^	fraction^c^	DB-FFAP	DB-1701	DB-5	FD factor^e^	earlier reported as volatile constituent of Gouda
**1**	ethyl butanoate	fruity	NBF	1045	851	810	128	3, 4, 5
**2**	3-methylbutanol	malty	NBF	1225	848	730	32	3, 4, 5, 10
**3**	2-isopropyl-3-methoxypyrazine^f^	earthy	NBF	1451	1147	1101	64	15
**4**	acetic acid	vinegar-like	AF	1468	784	n.d.	2048	4, 5, 7
**5**	(*Z*)-2-nonenal	fatty, green	NBF	1529	1253	1140	64	-
**6**	(*E*)-2-nonenal	fatty, green	NBF	1560	1276	1154	256	-
**7**	2-methylpropanoic acid	sweaty, fruity	AF	1589	958	n.d.	512	7, 10
**8**	butanoic acid	sweaty-rancid	AF	1649	982	n.d.	4096	6, 7, 10
**9**	2- and 3-methylbutanoic acid^g^	sweaty-cheesy	AF	1691	1033	n.d.	4096	6, 7, 10
**10**	pentanoic acid	sweaty	AF	1763	1079	n.d.	32	7
**11**	hexanoic acid	sweaty	AF	1874	1173	n.d.	16	4, 5, 6, 7, 10
**12**	2-phenylethanol	flowery	NBF	1952	1282	1051	512	7, 10
**13**	trans-4,5-epoxy-(*E*)-2-decenal^f^	metallic	NBF	2041	1547	1375	32	-
**14**	γ-nonalactone^g^	peach-like	NBF	2081	1586	1366	16	10
**15**	δ-decalactone^g^	peach-like	NBF	2203	1695	1467	16	7
**16**	4-ethyloctanoic acid	goat-like	AF	2240	1439	n.d.	512	-
**17**	3-hydroxy-4,5-dimethyl-2(5*H*)- furanone^f^	lovage-like	AF	2260	1357	1108	512	14
**18**	δ-decalactone^g^	coconut-like	NBF	2268	1739	1491	512	6, 7, 10
**19**	unknown	leather-like	NBF	2288	1567	n.d.	512	-
**20**	5-ethyl-3-hydroxy-4-methyl-2(5*H*)-furanone^f^	lovage-like	AF	2327	1435	1196	1024	14,15
**21**	γ -dodecalactone^g^	peach-like	NBF	2438	1914	1676	64	6, 7, 10
**22**	δ-dodecalactone^g^	peach-like	NBF	2484	1948	1710	512	6, 7, 10
**23**	2-phenylacetic acid	honey-like	AF	2575	1496	1283	1024	14
**24**	vanillin	vanilla-like	AF	2599	1648	1391	1024	-

a The compound was identified on the basis of a comparison with reference compounds using the following criteria: retention index on three different capillary columns, odor quality and odor threshold perceived at the sniffing port, mass spectra in the EI- and CI-mode.

b Odor quality perceived at the sniffing port.

c Fraction containing the compound after separation into acidic fraction (AF) and neutral-basis fraction (NBF).

d Linear retention index.

e Flavor dilution factor determined by AEDA on the FFAP column.

f No unequivocal mass spectrum was obtained. Identification was based on the remaining criteria given in footnote ^a^.

g Stereochemistry was not analyzed. n.d. = not determined.

For compound identification, odor qualities and odor intensities perceived at the sniffing port as well as retention indices on three different stationary phases were determined and compared to data available in a database consisting of odorants previously identified as key odorants in food.^41^ The suggested structure for the respective target compound was then verified by recording mass spectra of the respective reference compound in MS/EI and MS/CI modes.

Following this procedure, butanoic acid (**8**) and 2- and 3-methylbutanoic acid (**9**) were identified as compounds with the highest FD factor of 4096 in the AF, followed by acetic acid (**4**) with an FD factor of 2048 (Table 2). In addition, 5-ethyl-3-hydroxy-4-methyl-2(5*H*)-furanone (**20**, lovage-like), 2-phenylacetic acid (**23**, honey-like), and vanillin (**24**, vanilla-like) were determined with an FD factor of 1024. 2-Methylpropanoic acid (**7**, sweaty-fruity), 4-ethyloctanoic acid (**16**, goat-like), and 3-hydroxy-4,5-dimethyl-2(5*H*)-furanone (sotolon, **17**, lovage-like) showed a somewhat lower FD factor of 512.

In the NBF, the highest FD factor of 512 was found for 2-phenylethanol (**12**), δ-decalactone (**18**), and δ-dodecalactone (**22**). (*E*)-2-Nonenal (**6**, fatty, green) and ethyl butanoate (**1**, fruity) showed lower FD factors of 256 and 128, respectively (Table 2). Among the compounds characterized, five odor-active compounds, namely, (*Z*)-2-nonenal (**5**), (*E*)-2-nonenal (**6**), trans-4,5-epoxy-(*E*)-2-decena (**13**)l, 4-ethyloctanoic acid (**16**), and vanillin (**24**), were identified for the first time in a Gouda-type cheese.

### Quantitation of Selected Odor-Active Compounds in the Gouda Cheese Made from Pasteurized Milk (PM-G)

Sixteen aroma-active compounds showing high FD factors were selected for quantitation. Butane-2,3-dione and 3-methylbutanal were quantitated because a contribution of both compounds known as odorants of other cheeses seemed likely.^17,21^

The by far highest concentration was determined for acetic acid (**4**, 844 mg/kg), followed by butanoic acid (**8**, 59 mg/kg), 3-methylbutanoic acid (**9**, 26 mg/kg), 2-methylpropanoic acid (**7**,11 mg/kg), hexanoic acid (**11**,10 mg/kg), and 2-methylbutanoic acid (**9**, 4.0 mg/kg) (Table 3; batch 1). Furthermore, quite high amounts were also found for δ-dodecalactone (**21**, 2.4 mg/kg) and δ-decalactone (**18**, 1.6 mg/kg) as well as for 2-phenylacetic acid (**23**, 1.3 mg/kg) and butane-2,3-dione (1.3 mg/kg). On the other hand, only low concentrations were found for the fruity-smelling ethyl butanoate (**1**) and ethyl hexanoate (35 and 16 μg/kg, respectively).

**Table 3:** Concentrations of 16 Odor-Active Compounds in 30 Weeks Ripened Gouda Cheese Made from Pasteurized Milk (PM-G)

	concn. (μg/kg)		
	batch 1		
compound	mean^a^	range	batch 2^b^
acetic acid	843,501	788,790–898,212	1,394,525
butanoic acid	59,205	58,334–60,076	103,638
3-methylbutanoic acid	25,608	25,099–26,117	18,448
2-methylpropanoic acid	11,049	11,014–11,083	22,860
hexanoic acid	10,183	10,158–10,207	9175
2-methylbutanoic acid	3980	3901–4059	1928
δ-dodecalactone	2394	2372–2417	3596
δ-decalactone	1618	1610–1626	1687
2-phenylacetic acid	1295	1285–1305	1059
butane-2,3-dione	1266	1262–1270	2435
pentanoic acid	390	387–393	369
2-phenylethanol	217	213–221	275
3-methylbutanol	171	170–172	228
3-methylbutanal	93	91–95	211
ethyl butanoate	35	34–36	28
ethyl hexanoate	16	15–17	9.6

a Mean value of at least three samples. The standard deviation was below 10%.

b Batch 2 was produced with different batches of milk but using the same recipe one year after batch 1.

To confirm the consistency of the cheese production on the lab scale, the PM-G cheese was produced after 1 year from different batches of the ingredients but following the same procedure. The concentrations of the aroma compounds showed the same trend confirming that the procedure of cheese production was quite consistent (Table 3, batches 1 and 2).

Alewijn et al. had previously also reported the highest concentration for short-chain fatty acids in Gouda cheese, i.e., acetic acid (133 mg/kg), followed by butanoic acid (12 mg/kg), 3-methylbutanoic acid (11 mg/kg), hexanoic acid (3.4 mg/kg), and 2-methylpropanoic acid (2.6 mg/kg).^7^ By contrast, Dirinck and de Winne found lower concentrations of 516–696 μg/kg for 3-methylbutanoic acid, and van Leuven et al. also found lower amounts of 3-methylbutanoic acid (1.1 mg/kg), and considerably lower concentrations of 2-methylpropanoic acid (163 μg/kg) and 2-methylbutanoic acid (16 μg/kg).^6,10^ The authors also reported somewhat lower concentrations for δ-decalactone and δ-dodecalactone (331–590 μg/kg) as measured by us.

### Calculation of Odor Activity Values

To correlate the concentrations with the odor thresholds, subsequently, odor activity values (OAVs) were calculated using odor thresholds determined in oil.^39^ Deodorized sunflower oil has been shown to be an appropriate matrix for the calculation of OAVs for cheese odorants, e.g., in studies on the aroma of Emmentaler and Gruyere cheese.^16,21^ The odor thresholds of 2-methylpropanoic acid and 2-methylbutanoic acid in oil were newly determined in this study (Table 4).

**Table 4:** Odor Thresholds (OT) and Odor Activity Values (OAV) of 15 Key Aroma Compounds in Gouda Cheese Made from Pasteurized Milk (PM-G)

		OAV^b^	
compound	OT [μg/L]^a^	batch 1	batch 2^c^
acetic acid	124^d^	6802	11,246
3-methylbutanoic acid	22^d^	1164	839
butanoic acid	135^d^	439	768
butane-2,3-dione	4.5^e^	281	541
2-methylpropanoic acid	325^j^	34	70
δ-dodecalactone	120^e^	20	30
2-methylbutanoic acid	203^j^	20	10
3-methylbutanal	5.4^f^	17	39
δ-decalactone	120^g^	14	14
2-phenylacetic acid	186^h^	7	6
hexanoic acid	5400^e^	2	2
ethyl butanoate	28^f^	1	1
2-phenylethanol	211^d^	1	1
3-methylbutanol	225^i^	<1	1
ethyl hexanoate	40^f^	<1	<1

a Odor thresholds were determined in sunflower oil.

b OAVs were calculated by dividing the concentration by the odor detection threshold.

c Batch 2 was produced one year after batch 1.

d Data taken from ref (40).

e Data taken from ref (30).

f Data taken from ref (16).

g Data taken from ref (44).

h Data taken from ref (45).

i Data taken from ref (32).

j Odor thresholds were determined in this study.

The highest OAV in the cheese (Table 4; batch 1) was calculated for acetic acid (**4**, 6802), followed by 3-methylbutanoic acid (**9**,1164), butanoic acid (**8**, 439), and butane-2,3-dione (281). OAVs above 10 were reached by 2-methylpropanoic acid (**7**, 34), δ-dodecalactone (**21**,20), 2-methylbutanoic acid (**9**,20), 3-methylbutanal (17), and δ-decalactone (**18**, 14). Lower OAVs were calculated for 2-phenylacetic acid (**23**, 7), hexanoic acid (**11**, 2), ethyl butanoate (**1**, 1), and 2-phenylethanol (**12**, 1), while the concentrations of 3-methylbutanol (**2**) and ethyl hexanoate did not exceed their odor thresholds (Table 4). The data for batch 2 (Table 4) produced after one year followed the same trend compared to the data determined in batch 1.

### Aroma Recombination Experiments

**Figure 1:**
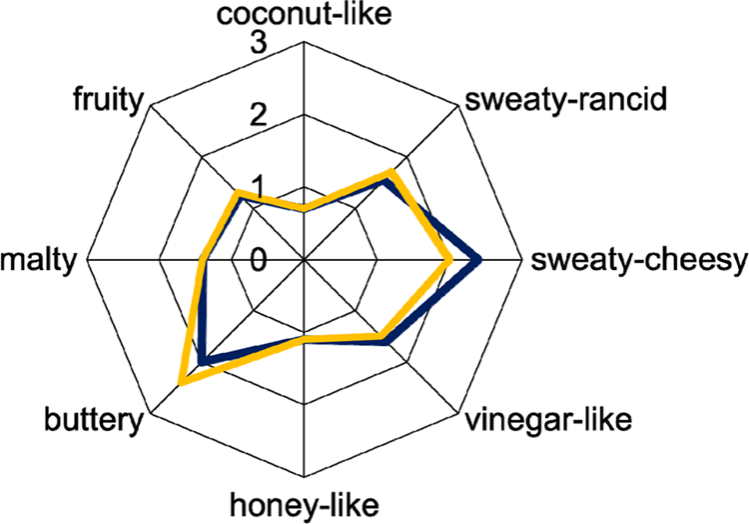
Comparative aroma profile analysis of 30 weeks ripened Gouda cheese made from pasteurized milk (blue) and the respective aroma recombinate (yellow).

An aroma recombinate was prepared by mixing the 13 odorants with OAVs ≥ 1(Table 4; batch 1) in their actual concentrations (Table 3). In addition, 3-methylbutanal was added to the recombinate. The odorant solution was blended with odorless Mozzarella powder, which had previously been proven to be an adequate cheese matrix for this purpose.^17,21^ First, a descriptive aroma profile analysis using eight odor attributes was carried out with a trained sensory panel. The aroma profiles showed a good agreement, except for a slightly stronger buttery attribute, in the recombinate and a more pronounced sweaty-cheesy attribute in the cheese (Figure 1). The overall similarity between the original Gouda cheese and the aroma model was evaluated with 2.5 on a scale from 1 to 3. One reason for the stronger perception of the buttery note in the recombinate is probably the low amount of butane-2,3-dione still present in the Mozzarella matrix,^13^ which could not be fully eliminated. This might also be the reason for the weaker sweaty-cheesy odor attribute, which was probably masked by the more pronounced buttery attribute. Another reason for the differences between the cheese aroma profile and the profile of the recombinate might be the different matrix, which is known to influence aroma release.

### Characterization of Odor-Active Compounds in Gouda Cheese Made from Raw (Nonpasteurized) Milk (RM-G)

**Figure 2:**
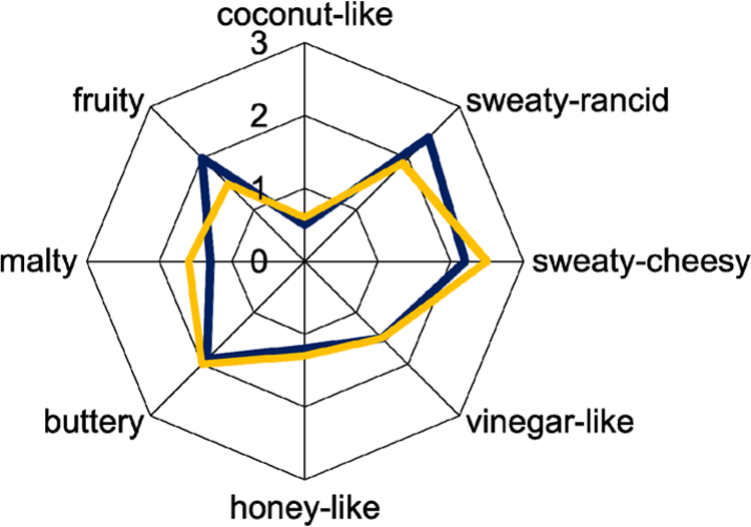
Comparative aroma profile analysis of 30 weeks ripened Gouda cheese made from nonpasteurized (blue) and pasteurized milk (yellow).

To clarify whether Gouda cheeses made from pasteurized and raw milk showed different aromas, first a comparative aroma profile analysis was done. The results revealed a more intensive fruity and sweaty-rancid aroma note in the cheese made from raw milk (RM-G) as compared to the cheese made from pasteurized milk (PM-G) (Figure 2). By contrast, the latter was characterized as more malty and sweaty-cheesy (Figure 2). An additional triangle test showed that both cheeses could clearly be differentiated with a significance level of 0.1% (data not shown).

### Identification of Odor-Active Compounds in the Gouda Cheese Made from Raw Milk (RM-G)

By HRGC/O evaluation, the same odor-active compounds were detected as in PM-G and, consequently, the same set of compounds was identified. However, the different FD factors suggested different concentrations in both types of cheeses (Table 5). For example, the fruity-smelling ethyl hexanoate (**2a**, Table 5) which was found in RM-G with an FD factor of 64 was not detectable in PM-G during GC/O (data not shown). Also, ethyl butanoate and 2-isopropyl-3-methoxypyrazine were found with higher FD factors in the RM-G while, for example, 3-hydroxy-4,5-dimethyl-2(5*H*)-furanone (sotolon) appeared with a higher FD factor in PM-G (Tables 2 and 5).

**Table 5:** Most Odor-Active Compounds (FD ≥ 16) in 30 Weeks Ripened Gouda Cheese Made from Raw Milk (RM-G)

			RI^c^			
no.	compound^a^	odor quality^b^	FFAP	DB-1701	DB-5	FD factor^d^
**1**	ethyl butanoate	fruity	1045	851	810	512
**2**	3-methylbutanol	malty	1225	848	730	16
**2a**	ethyl hexanoate	fruity	1245	1058	994	64
**3**	2-isopropyl-3-methoxypyrazine^e^	earthy	1451	1147	1101	512
**4**	acetic acid^f^	vinegar-like	1468	784	n.d.	4096
**5**	(*Z*)-2-nonenal	fatty, green	1529	1253	1140	128
**6**	(*E*)-2-nonenal	fatty, green	1560	1276	1154	512
**7**	2-methylpropanoic acid^f^	sweaty-fruity	1589	958	n.d.	512
**8**	butanoic acid^f^	sweaty-rancid	1649	982	n.d.	8192
**9**	2- and 3-methylbutanoic acid^f^	sweaty-cheesy	1691	1033	n.d.	4096
**10**	pentanoic acid^f^	sweaty	1763	1079	n.d.	64
**11**	hexanoic acid^f^	sweaty	1874	1173	n.d.	32
**12**	2-phenylethanol	flowery	1952	1282	1051	512
**13**	*trans*-4,5-epoxy-(*E*)-2-decenal^e^	metallic	2041	1547	1375	16
**15**	δ-decalactone^g^	peach-like	2203	1695	1467	32
**16**	4-ethyloctanoic acid^f^	goat-like	2240	1439	n.d.	512
**17**	3-hydroxy-4,5-dimethyl-2(5*H*)- furanone^e^,^f^	lovage-like	2260	1357	1108	128
**18**	δ-decalactone^g^	coconut-like	2268	1739	1491	256
**19**	unknown	leather-like	2288	1567	n.d.	512
**20**	5-ethyl-3-hydroxy-4-methyl-2(5*H*)-furanone^e^,^f^	lovage-like	2327	1435	1196	512
**21**	γ -dodecalactone^g^	peach-like	2438	1914	1676	64
**22**	δ-dodecalactone^g^	peach-like	2484	1948	1710	512
**23**	2-phenylacetic acid^f^	honey	2575	1496	1283	512
**24**	vanillin^f^	vanilla	2599	1648	1391	256

a Compound was identified on the basis of a comparison with reference compounds using the following criteria: retention index on three different capillary columns, odor quality and odor threshold perceived at the sniffing port, mass spectra in the EI- and CI-mode.

b Odor quality perceived at the sniffing port.

c Linear retention index.

d Flavor dilution factor determined by AEDA on the FFAP column.

e No unequivocal mass spectrum was obtained. Identification is based on the remaining criteria given in footnote ^a^.

f Compound was determined in the acidic fraction (AF).

g Stereochemistry was not determined. n.d. = not determined.

### Quantitation of Selected Odor-Active Compounds in Gouda Cheese Made from Raw Milk

Because the same set of odor-active compounds was found in both cheeses, the same 16 compounds were selected for quantitation.

Clear differences in the concentrations of most compounds were measured, and clear trends regarding the influence of the thermal milk treatment were observed (Tables 3 and 6). Although by far the most abundant aroma-active compound in both cheeses was acetic acid followed by butanoic acid, both acids were higher in the cheese from nonpasteurized milk (RM-G). In addition, higher amounts of the straight-chain fatty acids hexanoic acid and pentanoic acid were measured in RM-G. Also, the fruity-smelling esters ethyl butanoate and ethyl hexanoate were determined with clearly higher concentrations in RM-G (Tables 3 and 6), while the concentrations of δ-dodecalactone and δ-decalactone in both cheese varieties were nearly identical. By contrast, the branched-chain fatty acids 3- and 2-methylbutanoic acid showed clearly higher concentrations in PM-G. A calculation of odor activity values (Table 7) revealed the same four aroma compounds with the highest OAVs as in PM-G (Table 4); however, 3-methylbutanoic acid and butane-2,3-dione showed lower values, while butanoic acid was higher (Tables 4 and 7).

**Table 6:** Concentrations of 16 Odor-Active Compounds in 30 Weeks Ripened Gouda Cheese Made from Raw Milk (RM-G)

	concn.^a^ (μg/kg)	
compound	batch 1	batch 2^b^
acetic acid	909,719	1,680,038
butanoic acid	92,305	169,068
3-methylbutanoic acid	17,692	11,208
2-methylpropanoic acid	11,626	22,876
hexanoic acid	32,377	29,620
2-methylbutanoic acid	2286	1224
δ-dodecalactone	2507	3508
δ-decalactone	1593	1673
2-phenylacetic acid	1648	1372
butane-2,3-dione	437	696
pentanoic acid	657	732
2-phenylethanol	159	227
3-methylbutanol	106	150
3-methylbutanal	53	124
ethyl butanoate	131	88
ethyl hexanoate	154	90

a Mean value of at least three samples. The standard deviation was below 10%.

b Batch 2 was produced one year after batch 1.

**Table 7:** Orthonasal Odor Thresholds (OT) and Odor Activity Values (OAV) of 16 Key Aroma Compounds in Gouda Cheese Made From Raw Milk (RM-G)

		OAV^b^	
compound	OT [μg/L]^a^	batch 1	batch 2
acetic acid	124^c^	7336	13,549
3-methylbutanoic acid	22^c^	804	510
butanoic acid	135^c^	684	1252
butane-2,3-dione	4.5^d^	97	155
2-methylpropanoic acid	325^i^	36	70
δ-dodecalactone	120^d^	21	29
2-methylbutanoic acid	203^i^	11	6
3-methylbutanal	5.4^e^	10	23
δ-decalactone	120^f^	13	14
2-phenylacetic acid	186^g^	9	7
hexanoic acid	5400^d^	6	5
ethyl butanoate	28^e^	5	3
2-phenylethanol	211^c^	<1	1
3-methylbutanol	225^h^	<1	<1
ethyl hexanoate	40^e^	4	2

a Orthonasal odor thresholds determined in sunflower oil.

b OAVs were calculated by dividing the concentration of an odorant by its orthonasal detection threshold.

c Data taken from ref (36).

d Data taken from ref (26).

e Data taken from ref (12).

f Data taken from ref (37).

g Data taken from ref (38).

h Data taken from ref (28).

i Odor thresholds determined in this study.

For cheddar cheese, a similar trend for straight-chain fatty acids has been observed. Higher concentrations of butanoic acid as well as hexanoic acid were found in raw milk cheese compared to the same cheese made from pasteurized milk.^24,26^

The higher concentrations of the free straight-chain fatty acids suggested a pronounced lipolytic activity in the raw milk cheese. Therefore, the activity of lipases was determined, and a clearly higher lipolytic activity was measured in the raw milk Gouda cheese (Table 8).

**Table 8:** Comparison of Lipase Activity in Gouda Cheese Made from Pasteurized Milk (PM-G) and from Raw Milk (RM-G)

	lipase activity (U/h)^a^	
cheese-type	batch 1	batch 2
PM-G	1.9	1.4
RM-G	3.4	2.9

a 1 U/h = conversion of 1 mmol methylumbelliferone butanoate per h.

Lactones are assumed to be formed by an acid-catalyzed cyclization of the respective free hydroxy fatty acids. However, because the same amounts were found in the Gouda cheese from pasteurized and unpasteurized milk (Tables 3 and 6), obviously, lipolysis is not involved in lactone formation. Thus, the direct “lactonization” proposed by Alewijn et al.^9^ i.e., a nonenzymatic cyclization of hydroxy acids still bound to a triglyceride seems more probable as the mechanism for the formation of the lactones.

3-Methylbutanoic acid, 3-methylbutanal, and 3-methylbutanol are known to be formed by enzymatic degradation of the amino acid leucine.^42^ Because their amounts were higher in PM-G (Tables 3 and 6), these results suggested that this precursor amino acid is present in higher amounts in PM-G. This assumption was supported by the higher amounts of free leucine measured in this cheese type (Table 9). The same correlation was found for 2-methylbutanoic acid and its corresponding amino acid l-isoleucine as well as for 2-phenylethanol and l-phenylalanine. Both amino acids were also higher in PM-G (Table 9). For Cheddar cheese, Rehmann et al. also reported higher concentrations of 3-methylbutanol and 3-methylbutanoic acid as well as 2-methylbutanoic acid and 2-phenylethanol in a cheese made from pasteurized milk.^25,26^

**Table 9:** Concentrations of Selected Free Amino Acids in Gouda Cheese Made from Pasteurized Milk (PM-G) and from Raw Milk (RM-G)

	concn.^a^ (μg/kg)			
	batch 1		batch 2^b^	
amino acid	PM-G	RM-G	PM-G	RM-G
l-valine	4805	3438	3908	2475
l-isoleucine	1807	1099	1370	1080
l-leucine	4042	3354	4169	3131
l-phenylalanine	1844	1428	1647	1340

a Mean value of at least three samples. The standard deviation was below 10%.

b Batch 2 was produced one year after batch 1.

The quantitative differences in the aroma compounds found in the Gouda cheeses made from either pasteurized or raw, nonpasteurized milk can be explained by an enzymatic breakdown of precursors supplied by the milk following different biochemical activities of the bacterial populations. Because pasteurization inactivates almost the entire raw milk microorganisms, a growing population of “non-starter lactic acid bacteria” in the Gouda cheese made from raw milk is likely.^43^ A good indicator for such “non-starter lactic acid bacteria” is the presence of d-lactate, generated from l-lactate by the racemic activity of wild bacteria strains.^43^ To prove this assumption for our cheeses, d- and l-lactate concentrations in both Gouda varieties were measured (Table 10). As expected, a higher amount of d-lactate was detected in the RM-G cheese with a reduced amount of l-lactate (Table 10). Thus, the higher activity of non-starter lactic acid bacteria in RM-G was responsible for the differences in some key aroma compounds and, thus, the difference in the overall aroma profiles (Figure 2).

**Table 10:** Concentrations of l-Lactate and d-Lactate in Gouda Cheese Made from Pasteurized Milk (PM-G) and Raw Milk (RM-G)

	conc.^a^ (μg/kg)			
	batch 1		batch 2	
	PM-G	RM-G	PM-G	RM-G
l-lactate	1928	1510	1641	1397
d-lactate	31	506	74	727

a Mean value of at least three samples. The standard deviation was below 10%.

### Aroma Recombination of RM-G

An aroma recombinate of PM-G was prepared using the quantitative data reported in Table 6. On a scale from 0 (no similarity) to 3 (identical aroma profile), the recombinate was evaluated with a similarity of 2.6 compared to the RM-G cheese confirming that the key aroma compounds were identified (data not shown).

As found for the aroma profiles of both cheeses (Figure 2), a comparison of both aroma recombinates by aroma profile analysis confirmed the stronger sweaty-rancid and fruity attributes in the recombinate of the RM-G, while the sweaty-cheesy, buttery, and malty attributes were slightly more intense in the PM-G recombinate.

Application of the sensomics concept revealed clear differences in the key aroma compounds between 30 weeks ripened pilot-scale Gouda-type cheeses made from either pasteurized milk or raw milk. On the molecular level, in particular differences in the OAVs of straight-chain and methyl-branched fatty acids as well as esters and butane-2,3-dione are responsible for the different aroma profiles. Although the same set of key aroma compounds were elucidated in both types of Gouda cheese, the differences in the overall sensory perception are caused by different concentrations of key aroma compounds. During cheese ripening, the different set of microorganisms and consequently enzymes initiate a different metabolization of the milk fat by lipolysis and also a liberation of free amino acids from the milk proteins, thereby generating aroma compounds in different concentrations. To evaluate the influence of the ripening time on the aroma profiles of Gouda cheeses, further studies are on the way.

## Abbreviations

AEDA: aroma extract dilution analysis
AF: acidic fraction
NBF: neutral-basic fraction
OAV: odor activity value
PM-G: Gouda cheese made from pasteurized milk
RM-G: Gouda cheese made from raw (nonpasteurized) milk
SAFE: solvent-assisted flavor evaporation
SIDA: stable isotope dilution assay (analysis)
